# Prognostic role of serum neutrophil gelatinase-associated lipocalin in cardiac arrest patients: A prospective observational study

**DOI:** 10.1097/MD.0000000000027463

**Published:** 2021-10-08

**Authors:** Changshin Kang, Yong Nam In, Jung Soo Park, Yeonho You, Jin Hong Min, Wonjoon Jeong, Hong Joon Ahn, Yong Chul Cho, Seung Ryu

**Affiliations:** a Department of Emergency Medicine, Chungnam National University Hospital, Jung-gu, Daejeon, Republic of Korea; b Department of Emergency Medicine, College of Medicine, Chungnam National University, Jung-gu, Daejeon, Republic of Korea; c Department of Emergency Medicine, Chungnam National University Sejong Hospital, Sejong, Republic of Korea.

**Keywords:** acute kidney injury, biomarkers, neutrophil gelatinase-associated lipocalin protein, out-of-hospital cardiac arrest, prognosis, targeted temperature management

## Abstract

Accurate neurological prognostication is of the utmost importance to avoid futile treatments in patients treated with targeted temperature management (TTM) after out-of-hospital cardiac arrest (OHCA). This study aimed to investigate the prognostic value of serum neutrophil gelatinase-associated lipocalin (NGAL) by comparing with neuron-specific enolase (NSE), which is currently recommended by international guidelines in patients treated with TTM after OHCA.

The study included 85 comatose adult patients with OHCA who underwent TTM between May 2018 and December 2020. Serum NGAL and NSE were measured at 24-hour intervals until 72 hours after return of spontaneous circulation (ROSC). The primary outcome was their prognostic performance for poor neurological outcome at 3 months after OHCA.

Forty-nine patients (57.6%) had a poor neurological outcome; NGAL levels at all time points measured were significantly higher in these patients than in those with a good outcome (*P* < .01). NGAL showed lower maximal sensitivity (95% confidence interval [CI]) under a false-positive rate of 0% for the primary outcome compared with NSE (18.2% [95% CI 8.2–32.7] vs 66.7% [95% CI 50.5–80.4]). The combination of NGAL with NSE at 48 h showed the highest sensitivity (69.1% [95% CI 52.9–82.4]) and had the highest area under the curve (0.91 [95% CI 0.81–0.96]) for a poor outcome. The prognostic performance of NGAL alone was inadequate at all time points. However, NGAL combined with NSE at 24 and 28 hours after ROSC showed improved sensitivity compared to NGAL alone.

NGAL should be considered a supplementary biomarker in combination with NSE for prognostication in patients with OHCA treated with TTM.

## 1 Introduction

Despite the numerous medical studies and clinical efforts to improve the prognosis of cardiac arrest (CA) survivors with the successful return of spontaneous circulation (ROSC), achievement of a reduction in mortality rate and an acceptable neurological outcome remains challenging in this patient population.^[1]^ In a previously published report, only 8% of patients had good neurological outcomes after receiving post-CA care for out-of-hospital cardiac arrest (OHCA).^[2]^ Considering that most comatose patients with poor neurological outcomes are considered eligible for withdrawal of life-sustaining treatment (WLST), accurate neurological prognostication is of the utmost importance to avoid futile treatments in patients destined for a poor outcome and inappropriate WLST in patients who may have a chance of neurological recovery.^[1]^

For these reasons, there have been many attempts to develop methods for accurate and efficient neurological prognostication in CA survivors. In particular, various biomarkers have been investigated because of their advantages over other tools, such as the ability to obtain quantitative results and their likely independence from the effects of sedative agents.^[3]^ Moreover, acute kidney injury (AKI) after CA is associated with higher mortality, a poor neurological outcome, and a prolonged hospital stay.^[4,5]^ Several studies have reported that neutrophil gelatinase-associated lipocalin (NGAL) can facilitate the diagnosis of AKI in critically ill adult patients.^[6–8]^ Therefore, NGAL has been suggested as a potential biomarker for predicting the mortality risk and neurological outcomes in patients with CA.^[9–11]^

However, to the best of our knowledge, there has not been a serial comparison between NGAL and neuron-specific enolase (NSE), which is the prognostic biomarker recommended in the current international guidelines for post-CA care,^[12]^ when measured for the entire duration of care after CA. Although Kaneko et al reported that serum NGAL has a predictive value for the neurological outcome in patients with OHCA comparable with that of serum NSE, they only measured these biomarkers on days 1 and 2.^[9]^ We suggest that the analysis of biomarkers measured within 24 hours of ROSC might be insufficient to predict outcomes in patients with OHCA, considering that the median time to death of these patients has been reported to be 3.5 days (interquartile range [IQR] 1.2–9.3).^[13]^

Therefore, this study aimed to compare the usefulness of serum NGAL for prognostication in patients with OHCA with that of serum NSE during the entire period of targeted temperature management (TTM) after CA. Similarly, we investigated whether a combination of serum NGAL and NSE can improve the prognostic performance of NSE by comparing the neurological outcomes when each biomarker was used alone and when they were used together.

## 2 Materials and methods

### 2.1 Study design and population

This prospective observational study included comatose adult patients with OHCA who received TTM at Chungnam National University Hospital (CNUH) between May 2018 and December 2020. The study was approved by the Institutional Review Board of our institution (CNUH-2017-10-027). Informed consent was obtained from the patients’ designated decision-makers before enrollment. Patients for whom further treatment was declined by their next-of-kin and those with a known medical history of end-stage renal disease (ESRD) were excluded.

### 2.2 TTM protocol

Patients were managed according to a previously published TTM protocol^[14]^ whereby TTM is induced using ice packs, intravenous (IV) cold saline, and TTM devices, namely, Arctic Sun and Energy Transfer Pads (Bard Medical, Louisville, CO). A target temperature of 33°C is maintained for 24 hours and monitored using a bladder or esophageal probe. Upon completion of the TTM maintenance phase, patients are rewarmed at a rate of 0.25°C per hour to 37°C. Midazolam (0.05 mg/kg as an IV bolus followed by a titrated continuous IV infusion of 0.05–0.2 mg/kg/h) and cisatracurium (0.15 mg/kg as an IV bolus followed by an infusion of up to 0.3 mg/kg/h) are administered for sedation and to control shivering, respectively. All patients received standard intensive care according to our institutional intensive care unit protocol.

### 2.3 Data collection and clinical variables

The following information was recorded: patient age and sex, presence of a witness at the time of collapse, bystander cardiopulmonary resuscitation, first monitored cardiac rhythm, etiology of CA, no-flow and low-flow time from CA, total dose of epinephrine until ROSC, Glasgow Coma Scale score immediately after ROSC, time interval between ROSC and measurement of the initial NGAL value, and the Sequential Organ Failure Assessment (SOFA) score.

### 2.4 Measurement of NGAL and NSE

Serum NGAL and NSE levels were obtained from blood samples drawn by an arterial line at 24-hour intervals until 72 hours after ROSC (ie, NGAL_initial, 24, 48, and 72_ and NSE_initial, 24, 48, and 72_). The first NGAL and NSE measurements were obtained at the time of applying an external cooling device after several procedures for TTM, including electrocardiography, echocardiography, brain imaging, or resuscitation for sustained ROSC. NGAL and NSE values were measured using a UniCel DxC 880i chemical analyzer (Beckman Coulter Inc., Brea, CA) with the NGAL Test kit (BioPorto Diagnostics, Gentofte, Denmark) and an electrochemiluminescence immunoassay with Elecsys NSE (COBAS e801; Roche Diagnostics, Rotkreuz, Switzerland). The NGAL and NSE measurements were in the ranges of 25 to 3000 ng/mL and 0.1 to 300 ng/mL, respectively.

### 2.5 Outcomes

The primary outcome was the neurological outcome at 3 months after OHCA. The neurological outcomes were measured using the Glasgow-Pittsburgh cerebral performance category (CPC) scale, either by face-to-face interviews or structured telephone interviews.^[15]^ The interviews were conducted by an emergency physician who was well-versed with our protocols and blinded to each patient's prognosis and clinical data. Patients were classified into 5 categories based on the CPC score as follows: CPC 1 (good performance), CPC 2 (moderate disability), CPC 3 (severe disability), CPC 4 (vegetative state), and CPC 5 (brain death or death). Of these, CPC scores 3 to 5 indicated a poor neurological outcome.

### 2.6 Subgroup analysis

To avoid the confounding effect of death within 72 hours after ROSC, a subgroup analysis was performed to determine the association between NGAL and outcomes in patients who survived until 72 hours after ROSC.

### 2.7 Statistical analysis

Categorical variables are presented as the frequency and percentage and were compared using Fisher exact test. Continuous variables are presented as the mean and standard deviation or as the median and IQR depending on the normality of the data. Differences in NGAL values between the 2 groups divided based on neurological outcomes were assessed using the Student *t* test or the Mann–Whitney *U* test. Receiver-operating characteristic (ROC) curves were constructed to determine the prognostic value of NGAL in terms of the primary outcome. An ROC curve plots the sensitivity of a measure on the *y* axis and (1 - sensitivity) on the *x* axis and measures the overall accuracy of the test. The most important summary index of the ROC curve is the area under the ROC curve (AUROC). In this study, the AUROC values of the various combinations of measurements were estimated in 2 steps. First, a probability value was obtained by binary logistic regression analysis. Second, a ROC curve analysis was performed using this probability value as a test variable. The DeLong method was used to perform a pairwise comparison of the ROC curves for serum NGAL, NSE, and their combinations at each time point.^[16]^ The statistical analyses were performed using IBM SPSS Statistics (version 25.0; IBM Corp., Armonk, NY) and MedCalc version 15.2.2 (MedCalc Software, Mariakerke, Belgium). Results were considered statistically significant at *P* < .05.

## 3 Results

### 3.1 Patient characteristics

**Figure 1:**
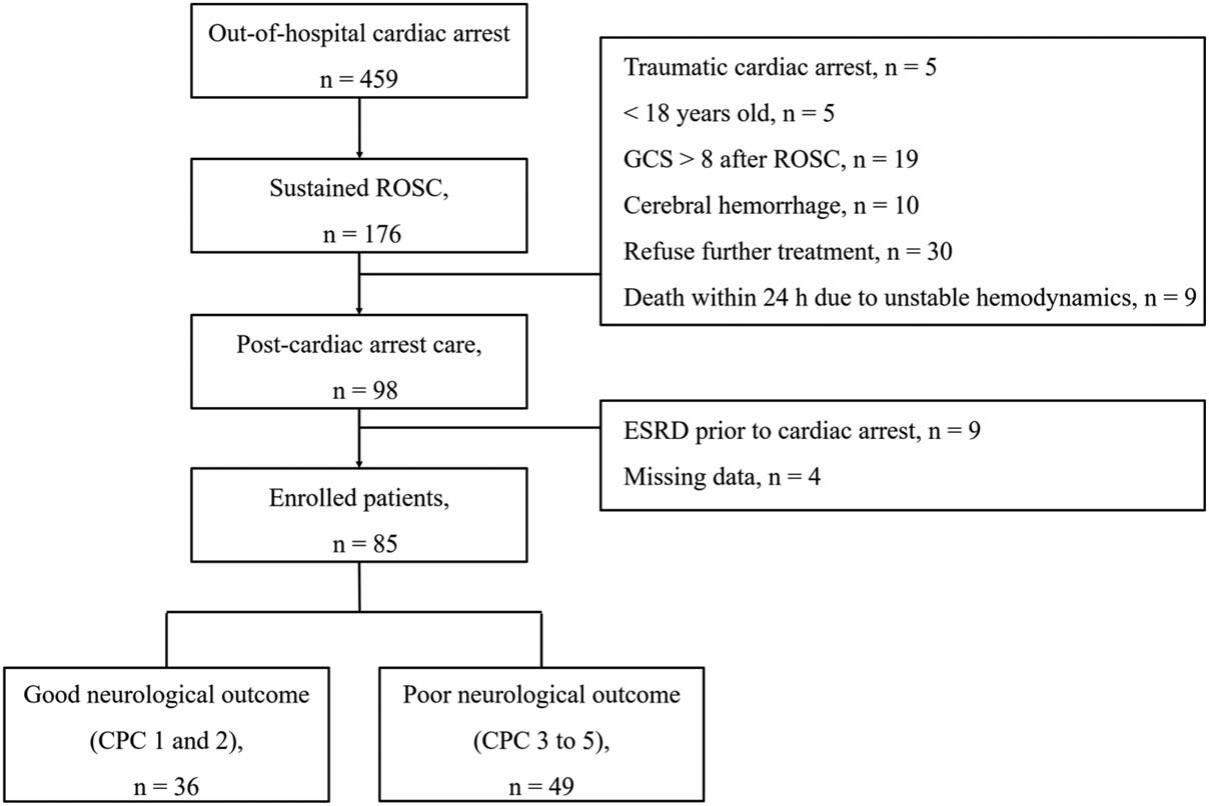
Diagram showing the flow of patients through the study. CKD = chronic kidney disease, ESRD = end-stage renal disease, GCS = Glasgow Coma Scale, ROSC = return of spontaneous circulation.

In total, 98 comatose OHCA patients were treated by TTM during the study period, 13 of whom were excluded because of a known medical history of ESRD (n = 9) or missed tests (n = 4); finally, 85 patients were included in the study. Forty-nine (57.6%) patients had poor neurological outcomes corresponding to a CPC score of 3 to 5 at 3 months after OHCA (Fig. 1). The basic characteristics of the enrolled patients are shown in Table 1. The group with a poor neurological outcome were significantly less likely to have had bystander cardiopulmonary resuscitation or have a cardiac etiology and shockable rhythm; rather, they had a significantly worse Glasgow Coma Scale score, significantly prolonged no-flow and low-flow times, and a significantly higher SOFA score (Table 1).

**Table 1:** Baseline demographics and clinical characteristics.

		Neurological outcome		
Variables	Total patients (n = 85)	Good neurological outcome (n = 36)	Poor neurological outcome (n = 49)	*P*
Age, y, mean ± SD	53.3 ± 18.4	53.3 ± 19.0	53.3 ± 18.1	.92
Sex, male, n (%)	62 (72.9)	28 (77.8)	34 (69.4)	.35
Cardiac arrest characteristics				
Witness, n (%),	53 (62.4)	27 (75.0)	26 (53.1)	.05
Bystander CPR, n (%)	60 (70.6)	31 (86.1)	29 (59.2)	<.01
Shockable rhythm, n (%)	24 (28.3)	20 (55.6)	4 (8.1)	<.01
Cardiac etiology, n (%)	32 (37.6)	24 (66.7)	8 (16.3)	<.01
No flow time, min, median (IQR)	2.0 (0.0–365.0)	0.5 (0.0–293.0)	9.0 (0.0–365.0)	<.01
Low flow time, min, median (IQR)	19.0 (2.0–68.0)	11.0 (2.0–30.0)	28.0 (2.0–68.0)	<.01
GCS immediately after ROSC, median (IQR)	3 (3–8)	3 (3–8)	3 (3–4)	<.01
Time to measure NGAL_initial_ from ROSC, h, median (IQR)	4.5 (1.2–19.5)	4.0 (1.2–10.4)	4.8 (2.2–19.5)	.05
Induction time at 33°C from ROSC, hours, median (IQR)	6.0 (2.0–19.0)	5.8 (2.6–12.8)	6.0 (2.0–19.0)	.32
SOFA score in ED, median (IQR)	10 (5–16)	8 (5–14)	11 (6–16)	.03

AKI = acute kidney injury, CAG = coronary angiography, CPR = cardiopulmonary resuscitation, CT = computed tomography, ED = emergency department, GCS = Glasgow coma scale, IQR = interquartile range, NGAL_initial_ = neutrophil gelatinase-associated lipocalin measured initially after ROSC, ROSC = restoration of spontaneous circulation, SD = standard deviation, SOFA = sequential organ failure assessment, TTM = target temperature management.

### 3.2 Comparison of NGAL and NSE values between groups

All the serum NGAL and NSE values obtained were significantly higher in the group with a poor neurological outcome (Table 2). A gradual decrease in NGAL was observed in both outcome groups after the peak level at baseline, whereas NSE peaked at 24 hours in the group with a good neurological outcome and at 72 hours in the group with a poor neurological outcome (Table 2).

**Table 2:** The serum concentration of neuron-specific enolase and neutrophil gelatinase-associated lipocalin.

		Neurological outcome		
Value, median (IQR)	Total patients, (n = 85)	Good neurological outcome, (n = 36)	Poor neurological outcome, (n = 49)	*P*
NGAL, ng/mL,				
NGAL_initial_ (n = 85)	163.8 (43.6–3000.0)	108.5 (43.6–1146.0)	256.3 (46.3–3000.0)	<.01
NGAL_24_ (n = 85)	149.2 (32.9–3000.0)	95.8 (32.9–1267.0)	238.4 (42.0–3000.0)	<.01
NGAL_48_ (n = 79)	129.8 (41.3–3000.0)	90.0 (41.9–1735.2)	186.0 (41.3–3000.0)	<.01
NGAL_72_ (n = 73)	111.4 (29.2–2194.8)	79.5 (29.2–1808.3)	126.2 (38.1–2194.8)	<.01
NSE, ng/mL				
NSE_initial_ (n = 85)	31.9 (8.5–300.0)	23.0 (8.5–54.8)	45.7 (15.6–300.0)	<.01
NSE_24_ (n = 85)	39.8 (12.8–300.0)	26.2 (12.8–85.3)	85.2 (16.6–300.0)	<.01
NSE_48_ (n = 77)	35.6 (9.3–300.0)	22.1 (9.3–64.2)	96.7 (11.5–300.0)	<.01
NSE_72_ (n = 73)	40.0 (8.0–300.0)	18.0 (8.0–144.0)	113.4 (12.4–300.0)	<.01

IQR = interquartile range, NGAL = neutrophil gelatinase-associated lipocalin measured at baseline (NGAL_initial_) = 24 (NGAL_24_) = 48 (NGAL_48_) = 72 hours (NGAL_72_) after return of spontaneous circulation; NSE = neuron-specific enolase measured at baseline (NSE_initial_), 24 (NSE_24_), 48 (NSE_48_), 72 hours (NSE_72_) after return of spontaneous circulation.

### 3.3 Prognostic performance of NGAL and NSE used alone and in combination

**Figure 2:**
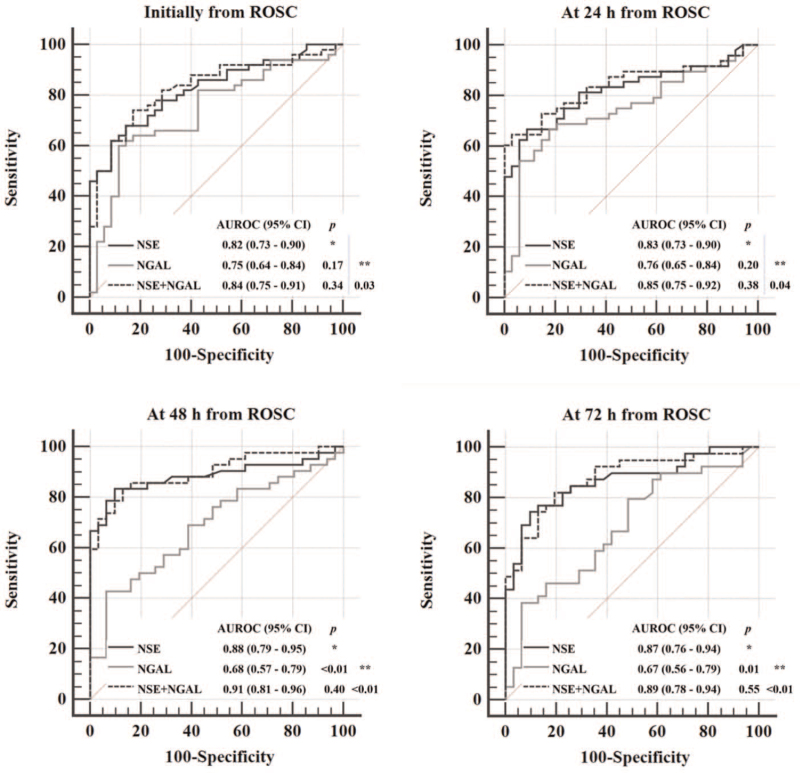
Statistical analysis of the areas under the receiver-operating characteristic curve for serum NGAL and NSE used alone and in combination. The DeLong test was used for statistical comparisons between (^∗^) serum NSE, serum NGAL, and a combination of the two biomarkers, and (^∗∗^) serum NGAL and a combination of the two biomarkers. NGAL = neutrophil gelatinase-associated lipocalin, NSE = neuron-specific enolase.

Figure 2 shows the AUROC values for each biomarker used alone and when used in combination. The AUROC values for the primary outcome were significantly higher at all time points measured after ROSC when the 2 biomarkers were used in combination than when NGAL was used alone (*P* = .03, *P* = .04, *P* < .01, and *P* < .01, respectively; Fig. 2), whereas the difference in these values between NSE and the combination did not reach statistical significance at any time point measured after ROSC (*P* = .34, .38, .40, and .55, respectively; Fig. 2). The AUROC values for NGAL decreased slightly over time, whereas those for NSE gradually increased. Thus, the AUROC values for NGAL measured at 48 hours and 72 hours after ROSC were significantly lower than those for NSE (*P* < .01 and *P* < 0.01, respectively; Fig. 2).

Table 3 shows the cut-off concentrations under a false-positive rate (FPR) of 0% and the corresponding sensitivity values for the 2 biomarkers when used alone and in combination. NGAL showed lower sensitivity than NSE for both primary outcomes at all time points (Table 3). The highest sensitivity values and confidence intervals (CIs) for the group with a poor neurological outcome were observed at 48 hours when the 2 biomarkers were used in combination (69.1% [95% CI 52.9–82.4]; Table 3). The combination of the 2 biomarkers numerically improved the sensitivity of NSE for the primary outcome at 24 hours and 48 hours after ROSC, albeit without reaching the threshold for statistical significance (Table 3).

**Table 3:** Prognostic performance of single and combined biomarkers.

	Poor neurological outcome				
	Cut-off	Sensitivity (95% CI)	Specificity (95% CI)	PPV	NPV (95% CI)
Biomarker alone, ng/mL					
NGAL_initial_ (n = 85)	1146.0	2.0 (0.1–10.0)	100.0 (90.3–100.0)	100	42.9 (41.9–43.8)
NGAL_24_ (n = 85)	1267.0	12.2 (4.6–24.8)	100.0 (90.3–100.0)	100	45.6 (43.0–48.2)
NGAL_48_ (n = 79)	1735.2	18.2 (8.2–32.7)	100.0 (90.0–100.0)	100	49.3 (45.8–52.8)
NGAL_72_ (n = 74)	1808.3	5.0 (0.6–16.9)	100.0 (89.4–100.0)	100	46.5 (44.7–48.3)
NSE_initial_ (n = 85)	54.8	44.9 (30.7–59.8)	100.0 (90.3–100.0)	100	57.1 (50.9–63.2)
NSE_24_ (n = 85)	85.3	49.0 (34.4–63.7)	100.0 (90.3–100.0)	100	59.0 (52.3–65.5)
NSE_48_ (n = 77)	64.2	66.7 (50.5–80.4)	100.0 (90.0 – 100.0)	100	71.4 (62.0–79.3)
NSE_72_ (n = 74)	144.0	43.6 (27.8–60.4)	100.0 (89.7–100.0)	100	60.7 (54.0–67.1)
Combinations					
NSE_initial__+_ NGAL_initial_		44.9 (30.7–59.8)	100.0 (90.3–100.0)	100	57.1 (50.9–63.2)
NSE_24_ + NGAL_24_		63.3 (48.3–76.6)	100.0 (90.3–100.0)	100	66.7 (58.1–74.3)
NSE_48__+_ NGAL_48_		69.1 (52.9–82.4)	100.0 (89.7–100.0)	100	72.3 (62.5–80.4)
NSE_72_ + NGAL_72_		43.6 (27.8–60.4)	100.0 (89.1–100.0)	100	59.3 (52.5–65.7)

CI = confidence interval, NPV = negative predict value, PPV = positive predict value, NGAL = 1neutrophil gelatinase-associated lipocalin measured at baseline (NGAL_initial_), 24 (NGAL_24_), 48 (NGAL_48_), 72 hours (NGAL_72_) after return of spontaneous circulation; NSE, neuron specific enolase measured at baseline (NSE_initial_), 24 (NSE_24_), 48 (NSE_48_), 72 hours (NSE_72_) after return of spontaneous circulation.

### 3.4 Subgroup analysis

Seventy-three patients remained alive at the end of TTM. The subgroup analysis without missing serum NGAL data is shown in Table 4. In the group with a poor neurological outcome, the AUROC values for NGAL_initial, 24, 48, and 72_ were 0.70 (95% CI 0.59–0.80), 0.71 (95% CI 0.59–0.81), 0.67 (95% CI 0.55–0.78), and 0.68 (95% CI 0.56–0.78), respectively (Table 4). Their sensitivity values with an FPR of 0% were 14.6% (95% CI 5.6–29.2), 9.8% (95% CI 2.7–23.1), 12.2% (95% CI 4.1–26.2), and 12.2% (95% CI 4.1–26.2), respectively (Table 4).

**Table 4:** Subgroup analysis of prognostic performance for poor neurological outcome in patients who fully measured NGAL for entire 72 hours of targeted temperature management.

Total patients, (n = 74)	AUROC (95% CI)	Cut-off	Sensitivity (95% CI)	Specificity (95% CI)	*P*			TP	FP	TN	FN	PPV	NPV
NGAL_initial_	0.70 (0.59–0.80)	1146.0	2.4 (0.1–12.9)	100.0 (89.4–100.0)	^∗^			6	1	32	35	100	44.6
NGAL_24_	0.71 (0.59–0.81)	1267.0	7.3 (1.5–19.9)	100.0 (89.4–100.0)	0.90	^∗∗^		4	1	32	37	80.0	46.5
NGAL_48_	0.67 (0.55–0.78)	1735.2	12.2 (4.1–26.2)	100.0 (89.1–100.0)	0.38	0.18	^∗∗∗^	5	1	32	36	83.3	47.1
NGAL_72_	0.68 (0.56–0.78)	1808.3	4.9 (0.6–16.5)	100.0 (89.4–100.0)	0.47	0.33	0.97	5	1	32	36	83.3	45.8

NGAL = neutrophil gelatinase-associated lipocalin measured at baseline (NGAL_initial_), 24 (NGAL_24_), 48 (NGAL_48_), 72 (NGAL_72_) h after return of spontaneous circulation; AUROC = the area under the receiver operating characteristic curve, CI = confidence interval, FN = false negative, FP false positive, NPV = negative predict value, PPV = positive predict value, TN = true negative, TP = true positive. References of DeLong test for statistical comparison between ^∗^NGAL_initial_ and the others, ^∗∗^NGAL_24_ and NGAL_48 or 72_, ^∗∗^ NGAL_48_ and NGAL_72_.

## 4 Discussion

This study demonstrated not only an association of the NGAL level with the neurological outcome at 3 months in patients who had OHCA treated with TTM but also showed that NGAL has meaningful prognostic value when used in combination with NSE. The sensitivity values with an FPR of 0% and AUROC values for the primary outcome were significantly lower for NGAL used alone than those when it was used in combination with NSE at all time points and when NSE was used alone at 48 hours and 72 hours from ROSC. Notably, when combined with NSE at 24 hours and 48 hours, NGAL numerically improved the sensitivity values when compared with NSE alone.

Brain injury is the major cause of death after ROSC in patients who with a history of CA. However, a significant proportion of these patients die of non-brain injury-related causes, such as cardiovascular shock and multiple organ dysfunction, during hospitalization.^[17–19]^ Therefore, using 1 brain-specific biomarker alone is insufficient for prognostication after CA,^[20]^ and NGAL has been identified as a possible additional prognostic biomarker of systemic stress after CA.^[7,9–11,21]^ Present guidelines for patients receiving post-CA care recommend that prognostication should have high specificity to avoid missing the opportunity for neurological recovery due to inappropriate WLST because of a perceived poor neurological prognosis.^[22]^ In a previous study, Jang et al reported that a combination of procalcitonin (a biomarker of extra-cerebral organ dysfunction) and S100 (a biomarker of cerebral damage) might improve prognostic performance, particularly sensitivity, over and above that of either biomarker when used alone.^[23]^ Our analysis using a combination of other biomarkers, NGAL and NSE, supports the notion of combining cerebral and extracerebral biomarkers to improve sensitivity. However, combining NGAL and NSE could not significantly improve the prognostic performance of NSE alone; hence, we suggest that this notion is exclusively valid for extracerebral biomarkers in patients with OHCA

To avoid the confounding effect of death within 72 hours after ROSC, we performed a subgroup analysis of all patients who survived until 72 hours after CA. However, the prognostic performance of NGAL measured at all time points for both outcomes had insignificant sensitivity of <15% with an FPR of 0%. This finding could be explained by the causes of mortality in patients with CA. Witten et al reported that the rate of death after OHCA due to the withdrawal of neurological care was higher than that after refractory hemodynamic shock (73% vs 17%).^[13]^ This report is consistent with our finding that 18 (60%) of the 30 nonsurvivors in our cohort died as a result of WLST or organ donation after brain death. NGAL, also known as lipocalin-2 (LCN2), is secreted by various tissues, such as kidney tubules, liver, lung, and gastrointestinal tract, at a low level in healthy controls.^[24]^ Similarly, its involvement in several pathways, such as apoptosis, bacteriostasis, renal tubule epithelial cell proliferation, and regeneration, has been reported.^[25]^ NGAL has several forms, including monomeric forms, dimers, and trimers. However, most NGALs are monomeric (with a molecular weight of 25 kDa) and are mainly produced by injured kidney tubule epithelium.^[26]^ NGAL levels increase rapidly during a proximal tubular injury and, thus, have emerged as a promising predictor of AKI.^[27]^ Further, NGAL represents a critical component of bacterial infectious crisis.^[6]^ Based on these reasons, NGAL is related to systemic inflammations rather than brain injury. Therefore, we suggest that NGAL is inappropriate as a single prognostic biomarker and should be considered as an additional modality for prognostication in patients with OHCA.

Determining when to obtain NGAL samples is important for the accurate prediction of the prognosis. Our results showed that the AUROC values for NGAL at 24 hours and 48 hours were relatively higher than those immediately and at 72 hours after ROSC. Additionally, the improvements in sensitivity using a combination of NGAL and NSE compared with using NSE alone were only observed at 24 hours and 48 hours after ROSC. Several studies have investigated the ability of NGAL to predict the clinical outcome after OHCA. Park et al. reported that NGAL measured at 72 hours was an optimal predictor of both mortality and neurological outcome.^[11]^ Another study found an association between the plasma NGAL level measured at 4 h after ROSC and a poor neurological outcome (adjusted odds ratio [OR], 1.004 [95% CI 1.001–1.007]).^[28]^ Moreover, Kaneko et al found that NGAL measured on day 2 (probably 24 hours after ROSC) had a predictive value comparable with that of NSE measured at the same time point for predicting the neurological outcome.^[9]^ However, in their multivariate regression analysis, Choi et al found that NGAL measured immediately and 3 h after ROSC was not a significant risk factor for a poor neurological outcome (NGAL_ROSC_: OR 1.017 [95% CI 0.998–1.036], *P* = .084; NGAL_3h_: OR 0.997 [95% CI 0.992–1.001], *P* = 0.113).^[7]^ Therefore, the optimal time to obtain NGAL samples for prognostication in patients with OHCA is controversial. Recently, recovery from AKI after CA has been observed in a significant proportion of these patients and can be a strong predictor of a good outcome.^[29,30]^ Moreover, Park et al reported that the duration of AKI after CA was 1 day in their group that recovered from AKI.^[2]^ Given that the best improvement of sensitivity obtained using a combination of 2 biomarkers was observed at 24 hours after ROSC, we suggest that NGAL can have significant prognostic performance in terms of predicting a poor neurological outcome at 24 hours after ROSC in patients with OHCA.

This study has several limitations. First, it was conducted at a single center and included a small number of patients. Therefore, the extent to which our findings are generalizable is unknown. Thirteen patients who underwent TTM during our study period were excluded because of a known history of ESRD or missing data, which could have introduced a degree of selection bias and further limited the generalizability of our results. Moreover, it has not yet been determined whether the serum NGAL level is elevated when kidney ischemia occurs in these patients. Therefore, the study results are not necessarily applicable to patients with ESRD. Therefore, larger studies are required to reduce the bias arising from small sample numbers. Another limitation is that NGAL levels before CA were not estimated in this study. Three patients in our cohort experienced CA due to hyperkalemia and one because of severe metabolic acidosis. All three patients with hyperkalemia died in the hospital (CPC 5), and the patient with metabolic acidosis survived with a poor neurological outcome (CPC 4). These patients may have had progressive kidney damage before CA, resulting in elevated serum NGAL levels at baseline. Nevertheless, further studies are required to investigate serum NGAL levels at baseline before CA.

## 5 Conclusions

NGAL was associated with the neurological outcome in patients with OHCA treated using TTM. However, its prognostic performance was not significant when used as a single biomarker. Therefore, NGAL should be considered as a supplementary biomarker in combination with NSE for prognostication in patients with OHCA. Nonetheless, further studies with a larger sample size and less selection bias are required to confirm our results.

## Author contributions

**Conceptualization:** Jung Soo Park.

**Formal analysis:** Yong Nam In, Wonjoon Jeong.

**Funding acquisition:** Jung Soo Park.

**Investigation:** Yong Nam In, Hong Joon Ahn.

**Methodology:** Changshin Kang, Yong Chul Cho.

**Software:** Changshin Kang.

**Supervision:** Yeonho You, Jin Hong Min.

**Visualization:** Changshin Kang, Jin Hong Min.

**Writing – original draft:** Changshin Kang, Jung Soo Park.

**Writing – review & editing:** Yong Nam In, Jung Soo Park, Seung Ryu.
